# A comparison of self-reported to cotinine-detected smoking status among adults in Georgia

**DOI:** 10.1093/eurpub/ckaa093

**Published:** 2020-06-26

**Authors:** Julianne Williams, Ivo Rakovac, Enrique Loyola, Lela Sturua, Nino Maglakelidze, Amiran Gamkrelidze, Kristina Mauer-Stender, Bente Mikkelsen, João Breda

**Affiliations:** c1 WHO European Office for Prevention and Control of Noncommunicable Diseases, Division of Noncommunicable Diseases and Health Through the Lifecourse, WHO Regional Office for Europe, Moscow, Russian Federation; c2 Noncommunicable Disease Department, National Center for Disease Control and Public Health, Tbilisi, Georgia; c3 Division of Noncommunicable Diseases and Health Through the Lifecourse, WHO Regional Office for Europe, UN City, Copenhagen, Denmark

## Abstract

**Background:**

Self-reported measures of tobacco use may have limited validity, particularly among some populations. This study aims to validate self-reported smoking measures among Georgian adults participating in the 2016 STEPS survey using cotinine biomarker measurements, and to explore potential differences according to sociodemographic characteristics. Additionally, this paper examines how the estimated prevalence of smoking in the population varies according to measurement type.

**Methods:**

Using the WHO standardized STEPS methodology, adults self-reported their smoking status. In a later stage of the survey, a subset of participants provided a urine sample, which was tested for cotinine. Using each participant’s objective cotinine measurement and their self-reported smoking status, we calculated the sensitivity, specificity and positive predictive value of self-reported smoking. Next, we calculated the estimated prevalence of smokers according to the type of measurement.

**Results:**

Results indicated high sensitivity (83.37%, 95% CI: 76.79–88.37%) among males and relatively low sensitivity (38.60% CI: 29.23–48.90%) among females. According to self-report, the prevalence of smokers was 26.44% (23.61–29.48%), while according to cotinine detection, the prevalence of smokers was 32.27% (29.16–35.55%). Among all subgroups, the self-reported prevalence of smoking was significantly lower than the cotinine-detected prevalence.

**Conclusions:**

To the best of our knowledge, this is the first time that the validity of the STEPS self-reported tobacco indicator has been tested. Self-reported measures of smoking status may lead to an under-estimation of smoking prevalence among Georgian adults, especially women. These findings suggest that integration of biochemical measures of smoking into tobacco use studies may be an important investment.

## Introduction

Smoking is a leading cause of preventable morbidity and pre-mature mortality worldwide, but particularly in the WHO European region, where the highest levels of tobacco-use prevalence (over 29%) have been reported.^1^ Smoking status can be detected using a variety of methods, including self-report. It is widely acknowledged, however, that people under-report exposure to behavioural risk factors, in a widely discussed phenomenon called ‘response bias’, which may occur when the study participants want to provide answers that are socially desirable.^2^ This ‘response’ bias is one reason why the accuracy of self-reported behaviours such as tobacco use may be less accurate among certain populations. For example, research indicates lower levels of accuracy related to self-reported tobacco use among pregnant women^3^ and among patients with respiratory diseases.^4^

Given that self-reports of smoking status may not always be reliable, a number of markers have been used to validate claims of non-smoking, including measures of cotinine in biological fluids.^5^^,^^6^ Cotinine, which is a major metabolite of nicotine and can be detected in blood, saliva and urine samples, is recognized as the most appropriate indicator of tobacco smoke exposure.^5–8^ Research to validate self-reported smoking behaviour using various methods (e.g. cotinine and thiocyanate) indicates that cotinine is the best method for determining smoking status in large-scale epidemiological studies.^9^ This use of biomarkers offers a more accurate way to systematically measure under-reporting of behaviours like tobacco use, as part of health surveillance, and if it is done well, it can help us to understand inequalities in reporting.

A report by Gorber et al.^5^ systematically reviewed the literature to measure concordance between self-reported smoking and smoking confirmed by cotinine measurement. Overall, the data showed trends of underestimation when smoking prevalence was based on self-report. It also found varying sensitivity levels for self-reported estimates depending on the population that was studied. The validity of self-reported tobacco use may vary according to sex ^10^ and the perceived social acceptability of smoking.^11^

Self-reported measures of tobacco use are frequently used in intervention and surveillance research. For example, self-report is used to assess tobacco use in the Stepwise approach for non-communicable disease (NCD) risk factor surveillance (STEPS), which is one of the main international surveys for collecting data about NCDs and their risk factors. Given that the validity of this self-reported tobacco use may vary according to individual and contextual factors, it is important to assess the validity among various subpopulations.^12^

This study aims to validate self-reported smoking measures among Georgian adults participating in the 2016 STEPS survey using cotinine biomarker measurements, and exploring potential differences according to sex, age and education level. It also aims to examine how the estimated prevalence of smokers in the population varies according to the type of measurement that was used. To the best of our knowledge, this is the first time that the validity of the STEPS self-reported smoking indicator has been tested using cotinine.

## Methods

The STEPS survey was carried out in Georgia from June 2016 to September 2017.^13^ A description of the STEPS surveillance background and methods is detailed elsewhere.^14^

### Self-reported smoking

In Step 1 of the survey, participants were asked for behavioural information, including questions about tobacco use. One question was: ‘Do you currently smoke any tobacco products, such as cigarettes, cigars or pipes’. Participants who replied ‘no’ were classified as ‘non-smokers’. Those who replied ‘yes’ were asked a second question: ‘Do you currently smoke tobacco products daily?’ If participants also replied ‘yes’ to this second question, they were classified as ‘daily smokers’. Participants who replied ‘yes’ to the first question but ‘no’ to the second question were classified as ‘occasional smokers’. Because the cotinine test detects tobacco consumption that has occurred within the previous two to three days, it was not possible to validate the accuracy of self-report among ‘occasional smokers’ (who could have either a positive or negative cotinine result, depending on the duration of time since their last use of tobacco). Therefore, all validation analyzes were conducted on ‘daily smokers’ only, and ‘occasional smokers’ were excluded from the main analyzes (analyzes including ‘occasional smokers’ are provided in the appendices).

### Cotinine

In a later stage of the survey (Step 3), participants provided biochemical measurements, including a urine sample. The urine samples were tested for cotinine within two days after collection using the COT Rapid Test Strip (Rapid Labs, Essex, UK). Following the recommendations from the test manufacturer, Urine was stored at room temperature in a sealed labelled pouch at 2–8°C for up to 48 h prior to assay. Before testing, the urine specimen reached room temperature. The test strip was immersed vertically into the urine specimen for 10–15 s and then placed on a non-absorbent flat surface. Results were read after 5 min. According to the test results, participants who had urine with a concentration ≥200 ng/ml were classified as ‘cotinine-detected smokers’ and participants with urine that had a concentration <200 ng/ml were classified as ‘cotinine-detected non-smokers’.

### Analyses

Sex was coded as a binary variable (male and female). Age was categorized into four groups: 18–29, 30–44, 45–59 and 60–69 years. Education was categorized into three groups: those that completed a secondary education or less, those that completed high school and those that completed college, university or a post-graduate degree.

Using each person’s objective cotinine measurement and their self-reported smoking status, we calculated the sensitivity (i.e. the self-reported measure’s ability to detect ‘true positives’, calculated as the probability that self-reported tobacco use will be positive when the cotinine results are positive), specificity (i.e. the self-reported measure’s ability to detect ‘true negatives’, calculated as the probability that self-reported tobacco use will be negative when the cotinine results are negative) and the positive predictive value (i.e. the probability that subjects with a positive self-reported tobacco response had positive cotinine results) according to demographic characteristics. Next, we calculated the estimated prevalence of smokers and non-smokers according to the type of measurement used.

Calculations were completed in Stata 14 and accounted for the study’s clustered, multi-stage sampling design, along with the age and sex distribution of the Georgian population in 2016.

## Results

In total, 4212 participants provided measures of self-reported tobacco use. Among these participants, around half (*n* = 1933 participants) provided laboratory results of urinary cotinine (Supplementary figure S1). After excluding the ‘occasional smokers’, the sample consisted of 1901 adults. Cross tabulations show the sample size according to cotinine-based smoking status and self-reported smoking status in table 1.

**Table 1:** Sample size, sensitivity and specificity of self-reported vs. cotinine-detected smoking

			Self-reported smoking status			Age- and sex-adjusted		
			Non-smoker	Smoker	Total	Sensitivity	Specificity	PPV
Cotinine-detected smoking status	Overall	Non-smoker	1427	29	1456	74.66%	96.53%	91.12%
		Smoker	142	303	445	(69.03–79.58)	(94.46–97.85)	(85.92–95.43)
		Total	1569	332	1901			
	Sex							
	Males	Non-smoker	216	23	239	83.37%	90.07%	91.17%
		Smoker	42	246	288	(76.79–88.37)	(83.73–94.11)	(85.34–94.83)
		Total	258	269	527			
	Females	Negative	1211	6	1217	38.60%	99.47%	90.67%
		Positive	100	57	157	(29.23–48.90)	(98.66–99.79)	(78.49–96.28)
		Total	1311	63	1374			
	Age							
	18–29	Negative	145	5	150	68.82%	94.17%	87.05%
		Positive	21	41	62	(54.18–80.47)	(84.81–97.90)	(68.35–95.43)
		Total	166	46	212			
	30–44	Negative	315	8	329	74.07%	96.20%	91.33%
		Positive	38	88	126	(64.17–82.00)	(91.76–98.29)	(81.79–96.11)
		Total	353	96	449			
	45–59	Negative	533	9	542	82.27%	97.46%	94.02%
		Positive	51	120	171	(75.75–87.34)	(94.73–98.79)	(87.95–97.14)
		Total	584	129	713			
	60–69	Negative	431	7	438	68.02	98.35	91.12%
		Positive	32	54	86	(56.64–77.60)	(96.46–99.24)	(81.75–95.91)
		Total	463	61	524			
	Education							
	Secondary school completed or less	Negative	336	4	340	69.14%	96.24%	87.17%
		Positive	36	52	88	(55.15–80.33)	(89.36–98.74)	(68.24–95.55)
		Total	372	56	428			
	High school completed	Negative	260	6	266	74.65%	96.18%	91.11%
		Positive	28	65	93	(61.26–84.57)	(88.75–98.77)	(75.13–97.20)
		Total	288	71	359			
	College, university or post-grad completed	Negative	761	15	776	69.14%	96.24%	93.01%
		Positive	74	169	243	(55.15–80.33)	(89.36–98.74)	(86.43–96.52)
		Total	835	184	1019			

Results indicated that the sensitivity of the self-reported tobacco measure (i.e. its ability to detect ‘true positives’) varied between sexes, with relatively high sensitivity (83.37%, 95% CI: 76.79–88.37%) among males and relatively low sensitivity (38.60% CI: 29.23–48.90%) among females. Sensitivity was highest (82.27%, 95% CI: 75.75–87.34%) among adults aged 45–59 years and lowest among those aged 60–69 years (68.02%, 95% CI: 56.64–77.60%). Results indicated that the specificity of self-report (i.e. its ability to detect ‘true negatives’) was relatively higher for women (99.47%, 95% CI: 98.66–99.79%) than it was for men (90.07%, 95% CI: 83.73–94.11%). The positive predictive value (the probability that those with a positive self-report truly were smokers according to lab tests) was 91.12% (85.92–95.43%) overall and relatively similar for men and women.

**Figure 1:**
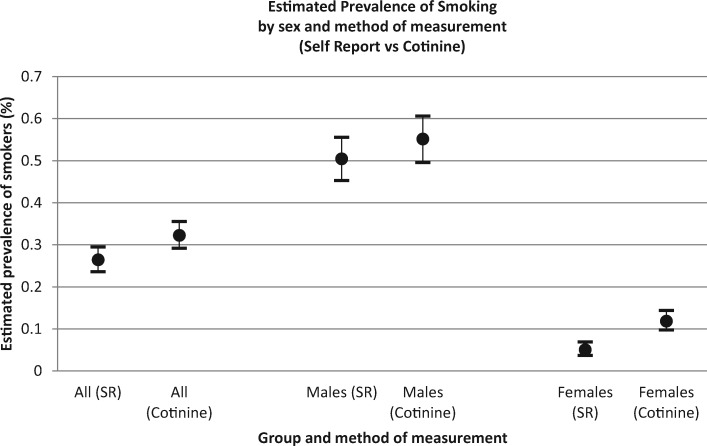
Estimated prevalence of smoking according to sex and method of measurement.

**Figure 2:**
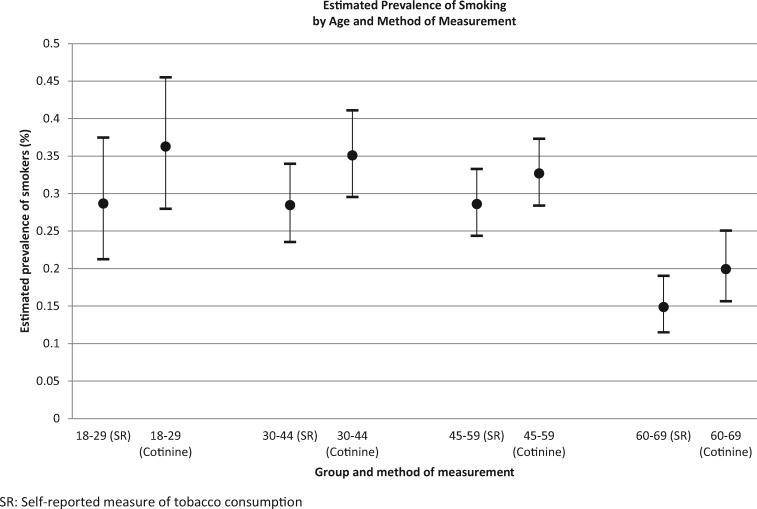
Estimated prevalence of smoking according to age and method of measurement.

Table 2 describes the prevalence of smokers and non-smokers according to the medium of measurement and stratified by sex, age and education level. Consistent with earlier studies, self-reported measures led to an under-estimation of smoking prevalence. According to self-report, the prevalence of smokers was 26.44% (23.61–29.48%), while according to cotinine detection, the prevalence of smokers was 32.27% (29.16–35.55%). Among all subgroups, the largest difference between self-reported and cotinine-detected smoking status was observed among women [a significant difference of 6.82% (5.01–8.63%)] (table 2 and figure 1) and among younger age groups (table 2 and figure 2). Among all subgroups, the self-reported prevalence of smoking was significantly lower than the cotinine-detected prevalence (table 2, figures 1 and 2 and Supplementary figure S2).

**Table 2:** Prevalence of smokers and non-smokers, by medium of measurement and sociodemographic characteristics (*n* = 1901)

	Self-reported status			Cotinine-detected status			Difference	
	%	Std. error (%)	95% CI (%)	%	Std. error (%)	95% CI (%)	Diff^a^(%)	95% CI (%)
Non-smoker	73.56	1.49	70.52–76.39	67.73	1.63	64.45–70.84		
Smoker	26.44	1.49	23.61–29.48	32.27	1.63	29.16–35.55	**5.83**	**3.56**–**8.10**
Males								
Non-smoker	49.55	2.63	44.40–54.71	44.83	2.82	39.36–50.43		
Smoker	50.45	2.63	45.29–55.60	55.17	2.82	49.57–60.64	**4.72**	**0.37**–**9.08**
Females								
Non-smoker	94.95	0.80	93.11–96.31	88.13	1.18	85.60–90.26		
Smoker	5.05	0.80	3.69–6.89	11.87	1.18	9.74–14.40	**6.82**	**5.01**–**8.63**
Non-smoker								
18–29 years	71.32	4.15	62.53–78.75	63.72	4.50	54.50–72.03		
30–44 years	71.53	2.66	66.02–76.46	64.89	2.95	58.90–70.45		
45–59 years	71.39	2.27	66.72–75.64	67.30	2.28	62.67–71.61		
60–69 years	85.12	1.91	80.95–88.50	80.06	2.39	74.94–84.35		
Smoker								
18–29 years	28.68	4.15	21.25–37.47	36.28	4.50	27.97–45.50	**7.60**	**0.62**–**14.58**
30–44 years	28.47	2.66	23.54–33.98	35.11	2.95	29.55–41.10	**6.63**	**2.39**–**10.87**
45–59 years	28.61	2.27	24.36–33.28	32.70	2.28	28.39–37.33	**4.09**	**1.70**–**6.47**
60–69 years	14.88	1.91	11.50–19.05	19.94	2.39	15.65–25.06	**5.05**	**2.10**–**8.01**
Non-smoker								
Secondary school completed or less	78.61	2.94	72.26–83.83	73.03	3.00	66.74–78.52		
High school completed	71.79	3.39	64.68–77.96	65.57	3.53	58.33–72.15		
College, university or post-grad completed	72.66	2.05	68.44–76.51	66.50	2.21	62.02–70.70		
Smoker								
Secondary school completed or less	21.39	2.94	16.17–27.74	26.97	3.00	21.48–33.26	**5.58**	**0.56**–**10.60**
High school completed	28.21	3.39	22.04–35.32	34.43	3.53	27.85–41.67	**6.22**	**0.76**–**11.68**
College, university or post-grad completed	27.34	2.05	23.49–31.56	33.50	2.21	29.30–37.98	**6.16**	**3.30**–**9.02**

Significant differences between self-report and cotinine-detected proportions are shown in bold.

^a^ Difference refers to the cotinine-detected proportion of smokers minus the self-reported proportion of smokers.

As a sensitivity analysis, we repeated all analyzes, but including ‘occasional smokers’ in the sample (*n* = 1933). The overall patterns were similar to the findings from analyzes with ‘daily smokers’ only (Supplementary tables S1 and S2).

## Discussion

This work suggests that self-reported measures of smoking status may lead to an under-estimation of smoking prevalence among Georgian adults, especially women. These findings suggest that continuing to integrate biochemical measures of smoking, such as cotinine, into tobacco use studies may be an important investment, particularly among specific subgroups, such as younger adults or women.

This paper is one of the first to examine the validity of self-reported tobacco measures in the STEPS surveys using cotinine measures, and to examine the validity of self-reported tobacco measures within adults in Georgia.

One question that emerges from this work is whether or not the 46% of participants who provided lab tests are representative of the larger study sample in terms of their smoking prevalence. While it is not possible to compare cotinine-detected smoking between the groups who provided lab tests and those who did not, it is possible to compare their self-reported smoking prevalence rates. Among the participants who did not provide lab tests (but who did provide self-report), the proportion of smokers was 31.06% (27.39–34.99%), which is higher than the self-reported proportion of smokers in the subsample with lab tests 26.44% (23.61–29.48%). This suggests that non-smokers may have been more likely to come in for the lab tests.

In contrast to our results, where the sensitivity of female self-reported tobacco use was low compared with males, Wong et al.^15^ found that sensitivity estimates were similar for males and females among a sample of 4530 Canadian adults. Similarly, del Carmen Valladolid-López et al.^12^ found that self-reported smoking validity indices were stable across genders. Consistent with our results, Hwang et al.^16^ found that the sensitivity of self-reported smoking in girls was much lower than it was in boys (43.5 and 67.0%, respectively). It is possible that these varying results may be due to differences between study populations in the social acceptability of female smoking. Our study adds to the body of evidence suggesting that the effect of sex on the accuracy of self-reported smoking is not consistent across study populations. This finding strengthens the argument that validation of self-reported smoking with cotinine or other biochemical methods may be a valuable addition to studies aiming to estimate the prevalence of smoking among the population.

At the same time, while cotinine-based assessment may have higher validity than self-report for assessing tobacco use, it also has some drawbacks. For example, compared with the non-invasive, inexpensive method of measuring self-reported tobacco consumption, measuring cotinine is relatively expensive and places an additional burden on respondents and data collectors.^17^ Furthermore, in studies such STEPS, which collect self-reported tobacco use on one day, followed by biochemical measures on another day, there is loss to follow-up with study participants for biochemical measurement, which means that compared with self-reported measures, the analyzes with biochemical measures have lower statistical power and larger error estimates, particularly for analyzes by subgroup (such as income or education). These factors suggest that it may not always be appropriate or feasible to measure tobacco exposure according to biomarkers.

In clinical settings, there has been an increased use of handheld devices to administer carbon monoxide (CO) tests, a method which involves asking a patient to exhale slowly into a device, which then provides a clinical with instant results that indicate recent levels of CO, a toxic gas found in tobacco smoke which binds to haemoglobin in red blood cells, and which can be a useful marker of regular smoking.^18^ Research indicates that smokers are more likely to make a successful quit attempt if a CO breath monitor is used as part of a supported and structured quit plan^19^ and in the UK, the National Institute of Clinical Excellence has included guidelines on use of the devices to assess smoking among pregnant women.^20^ The routine use of these devices within clinical settings may help normalize the testing of smoking (in the way that it is considered normal and expected to have a blood pressure test, or to have cholesterol measured), and this in turn, may help to reframe tobacco addiction as a disease rather than a choice.

As the usage of ‘electronic nicotine devices’ (ENDs) continues to rise, surveys must better account for the various sources of nicotine that study participants are using. In this study, e.g. it is possible that there were participants who were asked ‘Do you currently smoke any tobacco products, such as cigarettes, cigars, or pipes?’ and who reported ‘no’, but who were actually users of ENDS. In these instances, their ‘no’ response would have been justified, and therefore we may have been misrepresenting them as inaccurate self-reporters. To avoid this source of potential misrepresentation in the future, tobacco surveillance questions must better differentiate between traditional and novel tobacco products.

Researchers trying to identify whether or not to include the biochemical measures in their assessment of tobacco use may benefit from considering the social acceptability of smoking among specific subgroups. The social acceptability of smoking is declining in many Western countries.^21^ Research from Europe suggests that anti-tobacco information campaigns contribute to lower social acceptability.^22^ As anti-tobacco campaigns continue to run in these countries, it may be increasingly difficult to get an accurate self-report from respondents, making biochemical verification a valuable addition.

This paper explored differences in the accuracy of self-report according to sex, but it is also important to consider the ways that other key differences (e.g. socioeconomic status, ethnicity or urbanicity) may influence the accuracy of self-reported behaviours. For example, smoking prevalence in many populations is higher among lower socioeconomic groups, which may lead to differences in social norms related to smoking (e.g. increased acceptability), which may make a person feel more comfortable reporting themselves as smokers. However, the relationships are likely to be complex, and to vary between contexts. As another example, researchers have suggested that lower socioeconomic status individuals may receive social support, and may perceive disapproval from others they are spending money on tobacco products, and therefore, they might have *increased* social desirability bias.^23^ A study by Bryant et al. examined the accuracy of self-reported smoking among highly disadvantaged groups and indicated a strong agreement between their self-reported smoking status and blood CO-measured smoking status (with just over 6% of participants misclassified by self-report).^23^ The authors concluded that self-report may be valid in determining smoking status in low socioeconomic populations. However, this study was not based on a nationally-representative sample, and it did not compare the accuracy among individuals from a wide range of socioeconomic positions, which limits the generalizability of the findings.

Another study by Sheuermann et al. aimed to estimate the extent to which participant demographics were associated with the accuracy of self-report using salivary cotinine to verify self-report among 1178 participants enrolled in trials of hospital-initiated smoking cessation interventions.^24^ The study found that there were significant differences in accuracy according to education levels and race with higher levels of accuracy among highly educated participants and among white participants compared with lower educated or African–American participants.^24^ Although this study represented results from five different hospitals across the USA, it did not include findings from a wider, nationally-representative sample, and therefore there may be limited generalizability in the study findings. Future studies would benefit from exploring such differences among the general population, including differences according to income, levels of education, whether individuals live in urban or rural settings and ethnicity.

This study, along with many of the quantitative studies cited above, identified differences in self-report of smoking and biomarker-detected indicators of smoking, but they do not provide details into the reasons *why* some of these differences exist. Qualitative research has been conducted to identify differences in accuracy of self-report for a wide-range of health behaviours including diabetes control^25^ or sexual behaviour,^26^ and there is an opportunity to conduct similar work with smoking behaviour, in order to understand why specific groups may under-report, in order to improve our smoking surveillance methods. Having a more accurate understanding of the problem will improve our capacity to develop effective public health interventions to reduce tobacco-use within the population, and ultimately, to save lives.

## Supplementary Material

ckaa093_Supplementary_Data
