# A comparison of self-reported to cotinine-detected smoking status among adults in Georgia

**DOI:** 10.1093/eurpub/ckaa160

**Published:** 2020-08-20

**Authors:** Julianne Williams, Ivo Rakovac, Enrique Loyola, Lela Sturua, Nino Maglakelidze, Amiran Gamkrelidze, Kristina Mauer-Stender, Bente Mikkelsen, João Breda

*European Journal of Public Health*, 2020; doi:

In the above article, the following disclaimer was originally omitted in error: *The authors alone are responsible for the views expressed in this article and they do not necessarily represent the views, decisions or policies of the institutions with which they are affiliated*. This has been corrected online.

