# Supplementary material for: A comparison of self-reported to cotinine-detected smoking status among adults in Georgia
Source: Eur J Public Health. 2020 Jun 26;30(5):1007–12. doi: 10.1093/eurpub/ckaa093 (PMC7536257; doi:10.1093/eurpub/ckaa093)
Supplement: ckaa093_Supplementary_Data [file ckaa093_supplementary_data.zip › ckaa093-suppl_data/ejph-2019-07-om-0637-File004.docx]

Supplementary Figure 1. Study selection and participation flow chart.


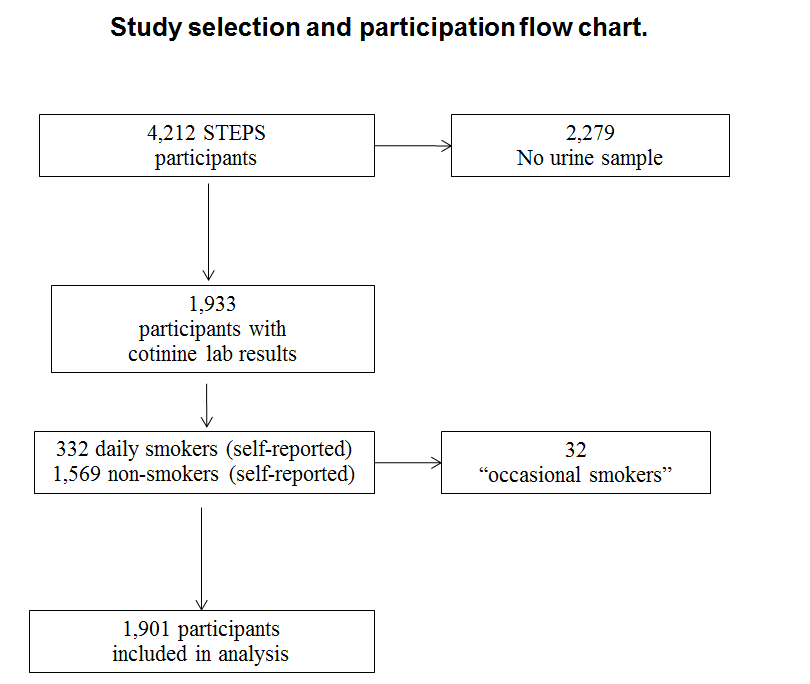


Supplementary Figure 2. Estimated prevalence of smoking according to education level and method of measurement

SR: Self-reported measure of tobacco consumption
