# Supplementary material for: A comparison of self-reported to cotinine-detected smoking status among adults in Georgia
Source: Eur J Public Health. 2020 Jun 26;30(5):1007–12. doi: 10.1093/eurpub/ckaa093 (PMC7536257; doi:10.1093/eurpub/ckaa093)
Supplement: ckaa093_Supplementary_Data [file ckaa093_supplementary_data.zip › ckaa093-suppl_data/ejph-2019-07-om-0637-File005.docx]

Supplementary Table 1. Sample size, sensitivity and specificity of self-reported vs cotinine-detected smoking, including ‘occasional smokers’

|  |  |  | **Self-reported smoking status** | | | **Age- and sex-adjusted:** | | |
| --- | --- | --- | --- | --- | --- | --- | --- | --- |
|  |  |  | **Non- smoker** | **Smoker** | **Total** | **Sensitivity** | **Specificity** | **PPV** |
| **Cotinine-detected smoking status** | **Overall** | **Non-smoker** | 1,427 | 43 | 1,470 | 75.98% | 95.15% | 88.58% |
|  |  | **Smoker** | 142 | 321 | 463 | (70.52, 80.71) | (92.91, 96.7) | (83.44, 92.28) |
|  |  | **Total** | **1,569** | **364** | **1,933** |  |  |  |
|  | **SEX** |  |  |  |  |  |  |  |
|  | Males | **Non-smoker** | 216 | 34 | 250 | 84.30% | 86.55% | 88.71% |
|  |  | **Smoker** | 42 | 257 | 299 | (77.99, 89.06) | (79.92, 91.24) | (82.98, 92.67) |
|  |  | **Total** | **258** | **291** | **549** |  |  |  |
|  | Females | **Negative** | 1,211 | 9 | 1,220 | 40.79% | 99.19% | 87.54% |
|  |  | **Positive** | 100 | 64 | 164 | (31.63, 50.64) | (98.22, 99.63) | (74.87, 94.30) |
|  |  | **Total** | **1,311** | **73** | **1,384** |  |  |  |
|  | **AGE** |  |  |  |  |  |  |  |
|  | 18-29 | **Negative** | 145 | 5 | 150 | 71.63% | 94.17% | 88.49% |
|  |  | **Positive** | 21 | 47 | 68 | (57.66, 82.39) | (84.81, 97.90) | (71.35, 95.95) |
|  |  | **Total** | **166** | **52** | **218** |  |  |  |
|  | 30-44 | **Negative** | 315 | 14 | 329 | 75.21% | 92.95% | 85.35% |
|  |  | **Positive** | 38 | 92 | 130 | (65.76, 82.74) | (87.70, 96.05) | (75.23, 91.79) |
|  |  | **Total** | **353** | **106** | **459** |  |  |  |
|  | 45-59 | **Negative** | 533 | 13 | 546 | 82.76% | 96.54% | 92.21% |
|  |  | **Positive** | 51 | 124 | 175 | (76.36, 87.71) | (93.60, 98.15) | (86.05, 95.79) |
|  |  | **Total** | **584** | **137** | **721** |  |  |  |
|  | 60-69 | **Negative** | 431 | 11 | 442 | 69.59% | 97.57% | 88.17% |
|  |  | **Positive** | 32 | 58 | 90 | (58.44, 78.82) | (95.50, 98.71) | (78.99, 93.66) |
|  |  | **Total** | **463** | **69** | **532** |  |  |  |
|  | **EDUCATION** |  |  |  |  |  |  |  |
|  | Secondary school completed or less | **Negative** | 336 | 7 | 343 | 73.34% | 95.23% | 86.66% |
|  |  | **Positive** | 36 | 57 | 93 | (59.87, 83.53) | (88.77, 98.05) | (70.95, 94.53) |
|  |  | **Total** | **372** | **64** | **436** |  |  |  |
|  | High school completed | **Negative** | 260 | 8 | 268 | 75.81% | 95.51% | 90.22% |
|  |  | **Positive** | 28 | 68 | 96 | (62.87, 85.30) | (88.66, 98.30) | (76.17, 96.38) |
|  |  | **Total** | **288** | **76** | **364** |  |  |  |
|  | College, university or post-grad completed | **Negative** | 761 | 20 | 781 | 73.34% | 95.23% | 86.66% |
|  |  | **Positive** | 74 | 176 | 250 | (59.87, 83.53) | (88.77, 98.05) | (70.95, 94.53) |
|  |  | **Total** | **835** | **196** | **1031** |  |  |  |

Supplementary Table 2. Prevalence of smokers and non-smokers, by medium of measurement and sociodemographic characteristics (n=1,933), including occasional smokers

|  |  | **Self-reported status** | | | | **Cotinine-detected status** | | | | **Difference*** | | |
| --- | --- | --- | --- | --- | --- | --- | --- | --- | --- | --- | --- | --- |
|  |  | **%** | **SE** | **95% CI** | | **%** | **SE** | **95% CI** | | **Diff*** | **95% CI** | |
| **Overall** | Non-smoker | 71.58% | 1.57% | 68.40% | 74.57% | 66.87% | 1.67% | 63.50% | 70.07% |  |  |  |
|  | Smoker | 28.42% | 1.57% | 25.43% | 31.60% | 33.13% | 1.67% | 29.93% | 36.50% | 4.71% | **2.38%** | **7.04%** |
| **Sex** | **Males** |  |  |  |  |  |  |  |  |  |  |  |
|  | Non-smoker | 47.15% | 2.67% | 41.95% | 52.42% | 44.39% | 2.79% | 38.99% | 49.93% |  |  |  |
|  | Smoker | 52.85% | 2.67% | 47.58% | 58.05% | 55.61% | 2.79% | 50.07% | 61.01% | 2.76% | -1.63% | 7.15% |
|  | **Females** |  |  |  |  |  |  |  |  |  |  |  |
|  | Non-smoker | 94.30% | 0.82% | 92.46% | 95.71% | 87.77% | 1.18% | 85.25% | 89.91% |  |  |  |
|  | Smoker | 5.70% | 0.82% | 4.29% | 7.54% | 12.23% | 1.18% | 10.09% | 14.75% | 6.53% | **4.72%** | **8.34%** |
| **Age** | **Non-smoker** |  |  |  |  |  |  |  |  |  |  |  |
|  | 18-29 years | 68.85% | 4.25% | 59.95% | 76.54% | 61.51% | 4.52% | 52.33% | 69.94% |  |  |  |
|  | 30-33 years | 68.86% | 2.68% | 63.36% | 73.87% | 64.65% | 2.95% | 58.65% | 70.23% |  |  |  |
|  | 45-59 years | 70.28% | 2.31% | 65.55% | 74.61% | 66.89% | 2.28% | 62.26% | 71.21% |  |  |  |
|  | 60-69 years | 83.72% | 2.01% | 79.37% | 87.31% | 79.38% | 2.42% | 74.21% | 83.74% |  |  |  |
|  | **Smoker** |  |  |  |  |  |  |  |  |  |  |  |
|  | 18-29 years | 31.15% | 4.25% | 23.46% | 40.05% | 38.49% | 4.52% | 30.06% | 47.67% | 7.33% | **0.59%** | **14.08%** |
|  | 30-33 years | 31.14% | 2.68% | 26.13% | 36.64% | 35.35% | 2.95% | 29.77% | 41.35% | 4.20% | -0.47% | 8.87% |
|  | 45-59 years | 29.72% | 2.31% | 25.39% | 34.45% | 33.11% | 2.28% | 28.79% | 37.74% | 3.39% | **0.92%** | **5.86%** |
|  | 60-69 years | 16.28% | 2.01% | 12.69% | 20.63% | 20.62% | 2.42% | 16.26% | 25.79% | 4.35% | **1.34%** | **7.35%** |
| **Education** | **Non-smoker** |  |  |  |  |  |  |  |  |  |  |  |
|  | Secondary school completed or less | 74.85% | 3.35% | 67.72% | 80.85% | 70.28% | 3.31% | 63.38% | 76.36% |  |  |  |
|  | High school completed | 70.30% | 3.39% | 63.23% | 76.52% | 64.66% | 3.51% | 57.49% | 71.23% |  |  |  |
|  | College, university or post-grad completed | 71.50% | 2.06% | 67.28% | 75.38% | 66.35% | 2.19% | 61.92% | 70.52% |  |  |  |
|  | **Smoker** |  |  |  |  |  |  |  |  |  |  |  |
|  | Secondary school completed or less | 25.15% | 3.35% | 19.15% | 32.28% | 29.72% | 3.31% | 23.64% | 36.62% | 4.57% | -0.36% | 9.49% |
|  | High school completed | 29.70% | 3.39% | 23.48% | 36.77% | 35.34% | 3.51% | 28.77% | 42.51% | 5.64% | **0.26%** | **11.03%** |
|  | College, university or post-grad | 28.50% | 2.06% | 24.62% | 32.72% | 33.65% | 2.19% | 29.48% | 38.08% | 5.15% | **2.11%** | **8.19%** |

*Difference refers to the cotinine-detected proportion of smokers minus the self-reported proportion of smokers. Significant differences between self-report and cotinine-detected proportions are shown in **BOLD**.
